# The Investigation of Perennial Sunflower Species (*Helianthus* L.) Mitochondrial Genomes

**DOI:** 10.3390/genes11090982

**Published:** 2020-08-24

**Authors:** Maksim Makarenko, Alexander Usatov, Tatiana Tatarinova, Kirill Azarin, Alexey Kovalevich, Vera Gavrilova, Renate Horn

**Affiliations:** 1 The Institute for Information Transmission Problems, 127051 Moscow, Russia; ttatarinova@laverne.edu; 2 Department of Genetics, Southern Federal University, 344006 Rostov-on-Don, Russia; usatova@sfedu.ru (A.U.); azarinkv@sfedu.ru (K.A.); kovalevich@sfedu.ru (A.K.); 3 Department of Biology, University of La Verne, La Verne, CA 91750, USA; 4 Vavilov Institute of General Genetics, 119333 Moscow, Russia; 5 School of Fundamental Biology and Biotechnology, Siberian Federal University, 660041 Krasnoyarsk, Russia; 6 The N.I. Vavilov All Russian Institute of Plant Genetic Resources, 190121 Saint Petersburg, Russia; v.gavrilova@vir.nw.ru; 7 Institute of Biological Sciences, Plant Genetics, University of Rostock, 18059 Rostock, Germany; renate.horn@uni-rostock.de

**Keywords:** perennial sunflowers, mtDNA, mitogenomes, mitochondrial mutations, *H. grosseserratus*, *H. strumosus*

## Abstract

The genus *Helianthus* is a diverse taxonomic group with approximately 50 species. Most sunflower genomic investigations are devoted to economically valuable species, e.g., *H. annuus*, while other *Helianthus* species, especially perennial, are predominantly a blind spot. In the current study, we have assembled the complete mitogenomes of two perennial species: *H. grosseserratus* (273,543 bp) and *H. strumosus* (281,055 bp). We analyzed their sequences and gene profiles in comparison to the available complete mitogenomes of *H. annuus.* Except for *sdh4* and *trnA-UGC*, both perennial sunflower species had the same gene content and almost identical protein-coding sequences when compared with each other and with annual sunflowers (*H. annuus*). Common mitochondrial open reading frames (ORFs) (*orf117*, *orf139*, and *orf334*) in sunflowers and unique ORFs for *H. grosseserratus* (*orf633*) and *H. strumosus* (*orf126*, *orf184*, *orf207*) were identified. The maintenance of plastid-derived coding sequences in the mitogenomes of both annual and perennial sunflowers and the low frequency of nonsynonymous mutations point at an extremely low variability of mitochondrial DNA (mtDNA) coding sequences in the *Helianthus* genus.

## 1. Introduction

From a genomic point of view, plants are unique organisms, since their cells have three genetic systems—nuclear, plastid, and mitochondrial—which function independently, but are simultaneously intimately interconnected. Nuclear genomes are of primary interest, while cytoplasmic genomes, especially mitochondrial, are often underestimated [1]. Mitochondrial DNA (mtDNA) in higher plants usually has complex multipartite structures, frequent insertions, and rapid rearrangements [2]. Mitochondrial genomes show significant variability not only between species but sometimes even within the same species [3]. Thus, even in species whose nuclear genomes have been investigated, the mitogenomic information is often lacking or incomplete [4].

The genus *Helianthus* is a diverse taxonomic group with approximately 50 species [5], divided into four sections: two annual sections, *Helianthus* and *Agrestis*, and two perennial sections, *Ciliares* and *Divaricati* [6]. Most sunflower genomic investigations are devoted to the economically valuable species, such as the annual species *H. annuus*, and in the case of the perennial species, *H. tuberosus*, while the other *Helianthus* species, especially perennial, are predominantly a blind spot. To the best of our knowledge, very few studies of perennial sunflower mtDNA are available [7]. Moreover, to date, the complete mitochondrial genomes of only one species—*H. annuus*—are deposited in GenBank. Mitochondrial DNA of cytoplasmic male sterility (CMS) sources, obtained from wild progenitors, may be considered as exceptions—for instance, the MAX1 CMS mitogenome [8] was gained through hybridization with *Helianthus maximiliani* Schrad as a maternal form. Since CMS systems are essential for the hybrid seed production of crops [9], particularly of sunflowers [10], the investigations of mitochondrial genomes of related wild species are also of interest for agricultural purposes.

As with *H. maximiliani*, the two species *H. grosseserratus* and *H. strumosus* represent perennial species and belong to the section *Divaricati*. *H. maximiliani* and *H. grosseserratus* are diploid, whereas *H. strumosus* can be either tetraploid (2n = 4x = 68) or hexaploid (2n = 6x = 102) [6]. The crossability between diploid species in the genus *Helianthus* is relatively good. However, while crossing tetraploid or hexaploid species is possible, there are considerable problems which require the application of biotechnological methods [11]. Many sunflower species, mostly diploid ones, have been used to develop new CMS systems for hybrid breeding in sunflowers, but very little is known about the general effects of different cytoplasms on sunflower performance. For instance, in field trials of Jan et al. [12] annual and perennial sunflower species were used to gain 20 cytoplasmic substitution lines, which were compared with the HA89 line. Although alloplasmic lines with cytoplasms of wild species showed predominantly neutral or negative effects on agronomic traits, such as an elevated level of lodging or reduced yield, the beneficial effects were also mentioned [12]. The characteristics of the achene walls are important for the industrial processing of sunflower seeds. *H. grosseserratus*, *H. maximiliani* and *H. strumosus* are characterized by a thin pericarp with a reduced sclerenchymatic layer, which represents an interesting trait in breeding for improved hullability [13].

In the current study, we assembled the complete mitogenomes of two perennial species: *H. grosseserratus* and *H. strumosus*. We analyzed their sequences and gene profiles in comparison to the available complete mitogenomes of *H. annuus* (MN175741.1, MN171345.1, MH704580.1, MG770607.2, MG735191.1, NC_023337.1, CM007908.1).

## 2. Materials and Methods

### 2.1. Plant Material and Mitochondrial DNA Extraction

The perennial species of *Helianthus*: *H. grosseserratus* Martens and *H. strumosus* L. (Figure S1) were obtained from the collection of the N. I. Vavilov All-Russian Institute of Plant Genetic Resources. Plant leaves were used for mitochondrial DNA isolation. For *H. strumosus*, the previously described method was used [14]. But for *H. grosseserratus*, we used a slightly different technique of multi-step centrifugations. but for *H. grosseserratus*, Leaves (5 g, without petiole and midrib) were homogenized by mortar and pestle in 20 ml of STE buffer (0.4 M sucrose, 50 mM Tris pH 7.8, 4 mM EDTA-Na2, 0.2% bovine serum albumin, 0.2% 2-mercaptoethanol) and then centrifuged using several steps: (1) 500 g for 5 min, picking the supernatant, (2) 4000 g for 5 min, picking the supernatant, (3) 10,000 g for 15 min, discarding the supernatant. The pellet was treated using 10 units of DNAse (Syntol, Moscow, Russia) for 7 min and then used for DNA isolation. For both samples, the DNA extraction was performed with a PhytoSorb kit (Syntol, Moscow, Russia), according to the manufacturer’s protocol.

### 2.2. Next-Generation Sequencing

Next-generation sequencing (NGS) libraries were made with a Nextera XT DNA Library Prep Kit (Illumina, San Diego, CA, USA), following the guidelines of Illumina. The quality and quantity of the libraries were evaluated with the Bioanalyzer 2100 (Agilent, Santa Clara, CA, USA) and with a Qubit 4 fluorometer (Thermo Fisher Scientific, Waltham, MA, USA). Sequencing was performed on different runs of NextSeq 500 (Illumina, San Diego, CA, USA) with the NextSeq 500/550 High Output Kit v2.5: 150 cycles for *H. grosseserratus* and 300 cycles for *H. strumosus.* A total number of 1,349,630 75-bp paired reads and 3,014,579 150-bp paired reads were generated.

### 2.3. Mitochondrial Genome Assembly and Annotation

For quality control of the reads, we used FastQC v 0.11.9 (https://www.bioinformatics.babraham.ac.uk/projects/fastqc/) and Trimmomatic v 0.39 software [15]. The generation of contigs was done with SPAdes Genome Assembler v 3.13.1 [16] using different k-mer values. The whole mitochondrial genome assembly was based on high coverage (depths > 50) contigs, using Bandage v 0.8.1 [17] program for visualizing de novo assembly graphs. The genome assembly was validated by remapping reads by Bowtie 2 v 2.3.5.1 [18] with visual revision of coverage uniformity (especially in the junctions of contigs) using Tablet v 1.19.09.03 [19]. Low coverage (depths < 15) regions of the *H. strumosus* mitochondrial genome were also verified by Sanger sequencing. The mitochondrial genomes were annotated with MITOFY [20], BLAST tool [21] and ORFfinder (https://www.ncbi.nlm.nih.gov/orffinder). Graphical genome maps were generated using the OGDRAW tool [22]. The annotated mitochondrial genomes of *H. grosseserratus and H. strumosus*, were submitted to the NCBI GenBank database (assigned accessions MT588180 and MT588181, respectively).

## 3. Results

First of all, it should be mentioned that different mitochondrial fraction isolation techniques showed significant variations in the amount of mtDNA derived sequences for the two perennial *Helianthus* species. In *H. strumosus*, only about 3.9% of the reads mapped to the mitogenome, whereas the NGS library of *H. grosseserratus* predominantly contained mitochondrial reads (67.3%). Such difference significantly influenced the complexity of mitogenome assembly. In the case of *H. grosseserratus*, a relatively small amount (1.3 mln) of short (75 bp) reads were obtained, and the assembly with a k-mer value equal to 65 generated only 14 high coverage (depths > 50) large contigs (N50 = 55,431) which allowed a quick scaffolding. In the case of *H. strumous*, twice as many elongated reads (3 mln, 150 bp) were observed, but many smaller contigs (N50 = 4,632), containing nuclear and chloroplast sequences, were present, which in turn increased the complexity of scaffolding. Additional filtering of mitochondrial contigs had to be performed in the case of *H. strumosus* mitochondrial genome assembly. Thus, for mitogenome assembly, the proportion of mtDNA in a sample is more crucial than the number of reads.

**Figure 1:**
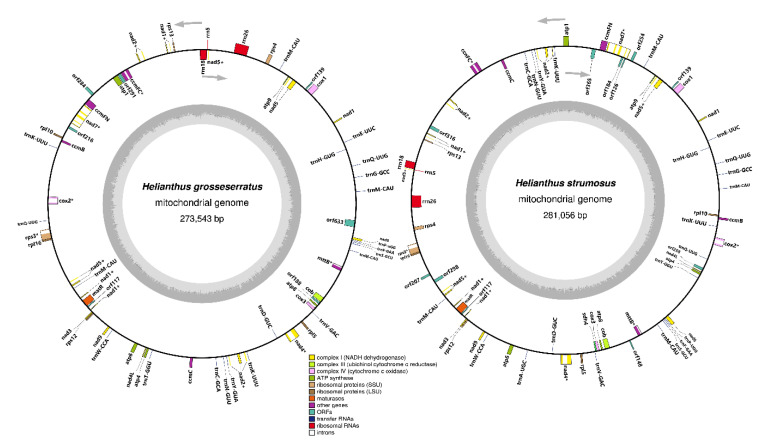
Mitochondrial genome maps of *H. grosseserratus* and *H. strumosus*. Arrows denote the gene’s transcription orientation. Intron-containing genes are marked by an asterisk (*) symbol and trans-spliced genes—by plus (+) symbol.

For both of the studied sunflower species, master circles of the mitochondrial chromosomes were gained (Figure 1), with lengths of 273,543 bp and 281,055 bp for *H. grosseserratus* and *H. strumosus*, respectively. Mitochondrial genomes often have a complex architecture, including sub-genomes or multichromosomal organization [23,24]. However, in the current study, we did not analyze the mtDNA stoichiometry and only focused on the master circle of the mitogenome type.

**Figure 2:**
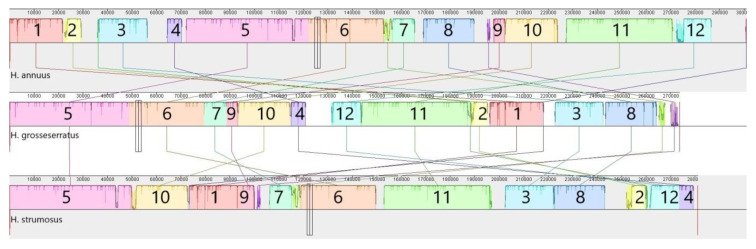
The progressive MAUVE alignment of *H. annuus*, *H. grosseserratus* and *H. strumosus* complete mitogenomes. According to sequence similarity, 12 syntenic blocks were identified, with a different order for each species.

The mtDNA alignment of perennial sunflower species with *H. annuus* allowed the identification of 12 syntenic blocks (>1 kbp, >98% similarity), as seen in Figure 2. Thus, the majority (>80%) of mitogenomes is similar for all three species, but there are also unique sequences. The orders of syntenic blocks vary significantly among species and may be considered as rearrangements. Nevertheless, there are common changes for perennial species, e.g., the adjunction of the 3rd and 8th blocks. Among other mtDNA structural differences, the variations in large repeat regions are of interest. In the *H. annuus* mitogenome, there are two large (>12.5 kbp) repeat regions, but in *H. grosseserratus*, these repeats are reduced to about 4.5 kbp, and in the case of *H. strumosus*—to about 1.1 kbp. The most prolonged repeat in *H. grosseserratus* comprises about 9.4 kbp, whereas in *H. strumosus* it counts only 7.1 kbp (Supplemental File 2).

The mitochondrial gene content was almost the same in the two perennial sunflower species, counting 31–32 protein-coding, three rRNA, 21–22 tRNA genes, and some open reading frames (ORFs). The mtDNA holds most genes that are typical for plant mitogenomes: NADH:ubiquinone oxidoreductase (Complex I) genes (*nad1*, *-2*, *-3*, *-4*, *-4L*, *-5*, *-6*, *-7*, and *-9*), *cob* (Complex III) genes, cytochrome c oxidase (Complex IV) genes (*cox1*, *-2*, and *-3*), ATP synthase (Complex V) genes (*atp1*, *-4*, *-8*, *and -9*), cytochrome c biogenesis genes (*ccmB*, *-C*, *-FC*, and *-FN*), *matR*, *mttB* and ribosomal protein genes (*rps3*, *-4*, *-12*, and *-13*, *rpl-5*, *-10*, and *-16*). In the mitogenomes of the two perennial sunflowers, succinate dehydrogenase (Complex II) genes are only represented by *sdh4* in *H. strumosus*, but in the case of *H. grosseserratus*, *sdh4* is a pseudogene, which has an internal stop codon. In the studied mitogenomes, nine intron-containing genes were identified. Five of the nine genes (*nad1*, *nad2*, *nad4*, *nad5*, and *nad7*) belong to mitochondrial complex I, and the remaining three genes were *ccmFC*, *cox2*, *mttB*, and *rps3*. The trans-spliced introns were observed in *nad1*, *nad2*, and *nad5*, which is consistent with angiosperm plants [25]. Other genes had a single cis-spliced intron, except *nad7* which counted four introns. The introns’ locations and splicing were similar to those observed in the mitochondrial genes of other flowering plants [26,27].

While comparing the amino acid sequences of the mitochondrial proteins of perennial sunflowers with annual sunflowers (NCBI accession MN171345.1), a limited number of differences could be detected (Table 1). The most severe changes concerned *atp6* and *nad6* in the mtDNA of *H. grosseserratus*. In *atp6* two nucleotide changes in the 37th codon resulted in the terminal leading to the N-terminal shortening of ATPase subunit 6, whereas in *nad6*, a frameshift led to a premature stop codon, resulting in C-terminal truncation of the encoded protein.

*H. grosseserratus* mitogenome includes 21 tRNA genes corresponding to 16 amino acids. The same tRNA genes are present in *H. strumosus* mtDNA, but an additional one (*trnA-UGC*) allows, in summary, to carry 17 amino acids by the mitogenome of *H. strumosus*. The in silico prediction of new open reading frames (ORFs) provided a large number of long (more than 300 bp) ORFs in both species. Meanwhile, we annotated only those (Table 2) with significant similarity to proteins and ORFs (according to the protein blast search) in sunflowers or other Magnoliophyta.

Among the discovered ORFs, we observed several interesting patterns. For instance, we discovered the presence of an ORF encoding the chloroplast-like ribosomal protein S11, in both currently studied species and all up-to-date, complete mitochondrial genomes of *Helianthus* in GenBank (MN175741.1, MN171345.1, MH704580.1, MG770607.2, MG735191.1, NC_023337.1, CM007908.1). Different ORFs—*orf284* (*H. grosseserratus*), *orf334* (*H. annuus*), and *orf365* (*H. strumosus*)—are encoding *psaA*-like proteins, which have almost identical amino acid sequences at the N-terminus. Chimeric mitochondrial genes, including *cox2* fragments, are often linked to male sterility inducing mtDNA of plants [28]. The *orf188* encoded protein contains 58 amino acids identical to the N-terminus of cytochrome oxidase subunit 2. While the *orf316*, present in *H. annuus* and *H. strumosus*, has 67 amino acids similar to the middle part of the COX2 sequence. The protein encoded by *orf188* in *H. grosseserratus* shares almost the same structure as the *cox2*-chimeric protein (QFS00065.1), which we identified in ANN2, a CMS line of sunflower in a previous study [29]. According to GenBank data, *orf316* is an ORF common to sunflower mtDNA. Notably, an uncharacterized protein from the cupredoxin superfamily (XP_022040088.1) annotated in the nuclear genome of *H. annuus* has a sequence equal to the *orf316* encoded protein. The largest ORF (*orf633*) was observed in the *H. grosseserratus* mitogenome. Most of the *orf633* encoded protein (397 aa) is identical to the N-terminus of ATP ATPase subunit 1.

## 4. Discussion

The mitochondrial genomes of perennial sunflower species are 7–9% smaller than annual sunflower mitogenomes. Meanwhile, except for the *sdh4* gene, annual and perennial sunflowers share the same protein-coding gene content. The *sdh3* and *shd4* genes are among the most “unstable” mitochondrial genes, with frequent cases of their transfer to the nucleus, and even related species may have different sets of succinate dehydrogenase genes [25]. The mitochondrial gene sequences showed slight differences between *H. grosseserratus* and *H. strumosus*, as well as between *H. annuus* and both perennial species. Only a few SNPs (1–3 per gene) leading to nonsynonymous substitutions occurred in *rps4*, *cob*, *rpl16*, *matR*, and *atp6*. Among the detected polymorphisms, only a single transversion (G to C) in the *atp6* gene (Table 1) was common for both perennial species in comparison to *H. annuus*. Notably, the same SNP in *atp6* was observed in the sunflower line with MAX1 (MH704580.1) CMS type, but was absent in PET1 (MG735191.1), PET2 (MG770607.2) CMS lines. Since the CMS lines have cytoplasmic genetic information (plastid and mitochondrial) initially obtained from wild species [30], PET1 and PET2 may be considered as *H. petiolaris* mitogenomes, and MAX1 as a *H. maximiliani* mitogenome. This SNP can likely be tracked only in perennial sunflowers, or at least those belonging to the *Divaricati* section. Sunflower species have unusually diverse karyotypes [31] and high rates of karyotypic evolution [32,33]. Within annual and perennial sunflower diploid species, there is a moderate variation in the sequence of plastid genes and quite a high variation in the nuclear genes [34]. Opposite to nuclear and plastid genes, an extremely low polymorphism level was found in sunflower mitochondrial genes. While only a few species were investigated, future research will clarify this feature. Similar conservation of mitogenome sequences can be observed in other species. Recently, a comparison of the domesticated lettuce (*Lactuca sativa*) mitogenome with two wild lettuce species (*L. saligna* and *L. serriola*) revealed identical sequences and rearrangements for *L. sativa* and *L. serriola*, but significant differences with the mitogenome of *L. saligna* [24]. Such data indicate that domestication had little influence on the mitochondrial genome as *L. serriola* is regarded as a wild ancestor of the domesticated lettuce [35].

Many mitochondrial genomes have abounded with foreign DNA acquired by horizontal transfer, especially from plastid genomes [36]. However, the functional activity of such plastid-originated insertions is of particular interest. The mitochondrial *rps11* gene is excluded from mtDNA in a vast number of eudicots [25,37]. The presence of *orf139*, encoding a protein that is similar to the chloroplast-like ribosomal protein S11, in the same position (close to *cox1* gene) of all up-to-date sunflower mitogenomes, does not seem accidental. The non-coding sequences in plant mitogenomes vary greatly and may rearrange [38], while the coding sequences are conservative [39,40], being under strong purifying selection [41]. Since *orf139* has an identical coding sequence in both perennial sunflowers and all up-to-date *H. annuus* mitogenomes (including CMS lines), we can assume that *orf139* plays a vital role in sunflower mitochondria. The functions of the ORFs similar to the *psaA* gene, are more cryptic. Three ORFs (*orf284*, *orf334*, and *orf365*) have a large identical part and have the same position in the mitogenome—between *atp1* and *ccmFN* (much closer to *ccmFN*). Thus, it is most likely that the chloroplast DNA (cpDNA) insertion entered the mitochondrial genome in a common sunflower ancestor, and then a limited number of mutations resulted in sequence divergence between these ORFs. Notably, cpDNA-derived sequences with partial homology to the *psaA* gene have been maintained over long periods in the mtDNA of different *Brassica* species [42].

Because of high mitochondrial DNA recombination rates, ORFs with partial mitochondrial gene sequences are common in mtDNA [40]. Such chimeric genes often cause the CMS phenotype in plants [28,43]. The *orf188* that we annotated in the *H. grosseserratus* mitogenome is similar to the previously described *cox2*-chimeric ORF (QFS00065.1), potentially playing a role in the formation of ANN2 CMS type in sunflower [29]. Nevertheless, the coding sequences of these two ORFs are not identical (about 76% similarity), but they have the same appearance in mtDNA. The other notable chimeric ORF in *H. grosseserratus* is *orf633*, which has no analogs, among other described sunflower mitogenomes. The *orf633* has a large part of sequence which is identical to the *atp1* gene, but its role is unclear. Studies have revealed the association between the CMS phenotype with mutations in *atp1* or a decrease in protein abundance [44]. However, to the best of our knowledge, there are no data about new atp1-chimeric ORFs involved in CMS. Since the subunit α (F1 sector) of ATPase (*atp1* encoded protein) lacks transmembrane domains, the association between additional rearranged *atp1* copy (*orf633*) and CMS phenotype is equivocal. Mitogenomes of higher plants are enriched with ORFs with an unknown function [45,46], while the CMS phenotype is often associated with unusual ORFs present in the mtDNA [47]. Thus, the current study may help to understand whether the ORFs discovered in future research will be new or standard for the sunflower mitogenomes.

## 5. Conclusions

The complete master circles of mitogenomes were obtained for *H. grosseserratus* (273,543 bp) and *H. strumosus* (281,055 bp). Except for *sdh4* and *trnA-UGC*, both perennial sunflower species had the same gene content and almost identical protein-coding sequences when were compared with each other or with the annual sunflower (*H. annuus*). Mitochondrial ORFs (*orf117*, *orf139*, *orf334*) common to sunflowers and unique ORFs for *H. grosseserratus* (*orf633*) and *H. strumosus* (*orf126*, *orf184*, *orf207)* were determined. The observed maintenance of plastid-derived DNA coding sequences in the mitogenomes of both annual and perennial sunflowers and the low frequency of nonsynonymous mutations point at an extremely low variability of mtDNA coding sequences in *Helianthus* genus. The current investigation may be useful for future studies in mitochondrial genomics.

## Figures and Tables

**Table 1:** Single nucleotide polymorphisms localized in protein-coding genes of perennial sunflower species.

Position in Mitogenome of *H. annuus*, (MN171345.1)	*H. annuus*	*H. grosseserratus*	*H. strumosus*	Gene	Substitution Type
37,865	T	T	**C**	*coxIII*	synonymous
43,358	G	**T**	G	*rpl5*	synonymous
67,110	C	T	C	*ccmB*	synonymous
112,693	T	**G**	T	*nad5*	synonymous
114,492	C	**T**	C	*atp9*	synonymous
122,169	C	C	**T**	*rps4*	nonsynonymous—Lys167Arg
122,990	T	**G**	T		nonsynonymous—Ile292Arg
169,209	A	A	**G**	*nad6*	synonymous
188,296	G	G	**T**	*cob*	synonymous
188,466	C	C	**A**		nonsynonymous—Asn42Lys
188,475	C	**A**	C		nonsynonymous—Phe45Leu
189,199	A	**C**	A		nonsynonymous—Ile287Leu
230,114	A	**C**	A	*rpl16*	nonsynonymous—Lys32Gln
250,522	G	**A**	G	*matR*	nonsynonymous—Ala458Glu
251,624	C	C	**T**		nonsynonymous—Gly91Arg
269,036	T	**G**	T	*atp6*	nonsynonymous—Phe37Ter
269,037	T	**A**	T		
269,064	G	**C**	**C**		nonsynonymous—Lys46Asn
269,155	T	T	**C**		nonsynonymous—Tyr77His

In bold—changes compared to *H. annuus.*

**Table 2:** ORFs annotated in *H. grosseserratus* and *H. strumosus* mitogenomes. The ORFs are named by their amino acid length. *orf148*—present in *H. annuus* with PET2 CMS type, *orf188*—similar ORF presented in *H. annuus* with ANN2 CMS type.

Name	Presence in			BLASTp Similarity
	*H. annuus*	*H. grosseserratus*	*H. strumosus*	
*orf117*	+	+	+	hypothetical protein
*orf126*	−	−	+	hypothetical protein
*orf139*	+	+	+	*rps11*
*orf148*	±	−	+	hypothetical protein
*orf184*	−	−	+	hypothetical protein
*orf188*	±	+	−	*cox2*
*orf207*	−	−	+	RNA polymerase
*orf254*	+	−	+	hypothetical protein
*orf259*	+	−	+	hypothetical protein
*orf* *291*	+	−	+	hypothetical protein
*orf298*	+	−	+	hypothetical protein
*orf316*	+	−	+	*cox2*
*orf334*	*orf334*	*orf284*	*orf365*	*psaA*
*orf633*	−	+	−	*atp1*

+ = present, − = absent, ± present in some accessions.

## Supplementary Materials

The following are available online at https://www.mdpi.com/2073-4425/11/9/982/s1, Figure S1: the appearance of perennial sunflowers, Table S1: large (>200 bp) repeats in mitogenomes of perennial sunflowers.
