# Supplementary material for: The Investigation of Perennial Sunflower Species (Helianthus L.) Mitochondrial Genomes
Source: Genes (Basel). 2020 Aug 24;11(9):982. doi: 10.3390/genes11090982 (PMC7565312; doi:10.3390/genes11090982)
Supplement: Supplementary file 1 [file genes-11-00982-s001.zip › Suppl 1.pdf]

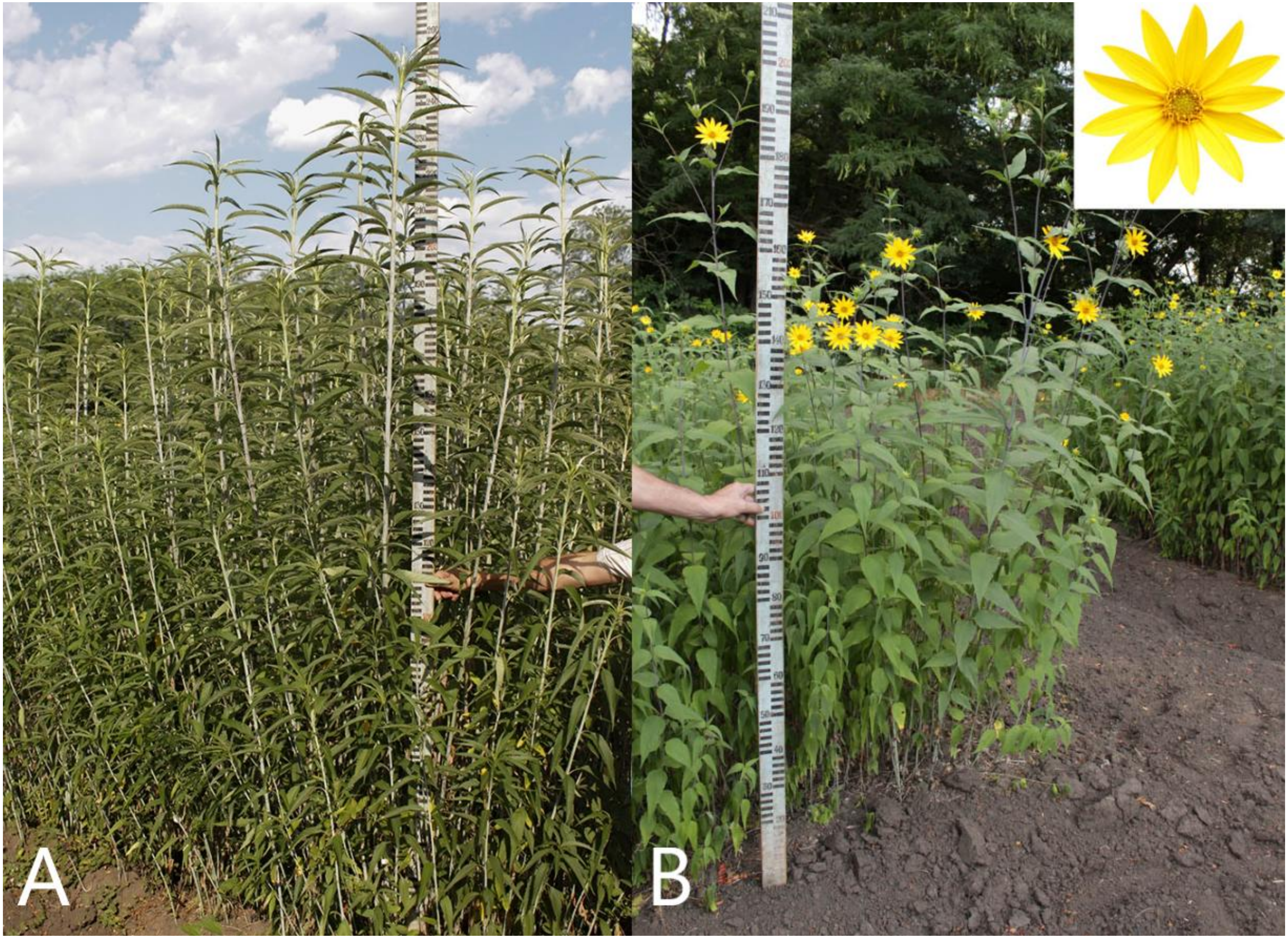

The appearance of perennial sunflowers: A – *H. grosseserratus*, B – *H. strumosus*.
The scale of the ruler is 10 cm
