# Supplementary material for: The Investigation of Perennial Sunflower Species (Helianthus L.) Mitochondrial Genomes
Source: Genes (Basel). 2020 Aug 24;11(9):982. doi: 10.3390/genes11090982 (PMC7565312; doi:10.3390/genes11090982)
Supplement: Supplementary file 1 [file genes-11-00982-s001.zip › Table S1.pdf]

Repeat length (bp)	Start	End	Start	End	Strand
			H. grosseserratus		
9440	120729	130168	217837	227278	Plus/Plus
5890	94082	99971	267654	273543	Plus/Plus
450	162097	162546	260246	260695	Plus/Plus
328	136738	137065	243029	243356	Plus/Plus
250	93553	93802	242143	242392	Plus/Plus
234	42203	42436	242393	242626	Plus/Plus
			H. strumosus		
7149	243490	250638	102950	110099	Plus/Plus
1997	271494	273490	71534	73530	Plus/Plus
450	239242	239691	171183	171632	Plus/Plus
309	149181	149489	50058	49750	Plus/Minus
297	221785	222081	71033	70737	Plus/Minus
210	260943	261152	153147	152938	Plus/Minus
215	260551	260765	194718	194503	Plus/Minus
203	269838	270040	118529	118327	Plus/Minus
